# Solitary Cutaneous Nodule as the Initial Presentation of Chronic Lymphocytic Leukemia/Small Lymphocytic Lymphoma

**DOI:** 10.7759/cureus.99859

**Published:** 2025-12-22

**Authors:** Brian A Moreno, Moises Lutwak, Eduardo Weiss, Saleeby Eli, Stanley Skopit

**Affiliations:** 1 Dermatology, Lake Erie College of Osteopathic Medicine, Bradenton, USA; 2 Dermatology, Larkin Community Hospital, South Miami, USA; 3 Dermatology, Hollywood Dermatology and Cosmetic Specialists, Hollywood, USA; 4 Dermatology, Skin and Cancer Associates Center for Cosmetic Enhancement, Aventura, USA

**Keywords:** case report dermatology, clinical dermatology, complex dermatology, dermatology consult, dermatology diagnosis, dermatology screening, dermatology teaching, medical dermatology, operational dermatology, skin disease/dermatology

## Abstract

Chronic lymphocytic leukemia/small lymphocytic lymphoma (CLL/SLL) is an indolent mature B-cell neoplasm that may rarely involve the skin, where it can clinically mimic primary cutaneous B-cell lymphomas (PCBCLs). When the skin is involved, lesions often appear as erythematous or violaceous papules, plaques, or nodules on the head, neck, or trunk. An 85-year-old male presented for evaluation of slowly enlarging, asymptomatic nodules on the right upper back and left posterior shoulder that had been present for many years. A shave biopsy of the right upper back lesion revealed a nodular dermal infiltrate of small lymphocytes. Immunohistochemistry showed CD20, CD79a, CD23, CD5, and BCL2 positivity with low Ki-67, consistent with CLL/SLL.

A second-opinion review at the National Institutes of Health confirmed the diagnosis and identified low-level marrow involvement by CLL/SLL. Whole-body positron emission tomography (PET) showed no hypermetabolic lymphadenopathy or visceral disease. The patient underwent excision of the back nodule with complex primary closure, and follow-up examinations have shown a well-healed scar without local recurrence. This case illustrates how CLL/SLL can present with a solitary cutaneous nodule and underscores the importance of histopathology and immunophenotyping in distinguishing secondary cutaneous involvement by systemic lymphoma from PCBCLs, as management and prognosis differ.

## Introduction

Primary cutaneous B-cell lymphomas (PCBCLs) are a group of extranodal non-Hodgkin lymphomas that present in the skin without evidence of systemic disease at diagnosis. They comprise several clinicopathologic entities, including primary cutaneous follicle center lymphoma (PCFCL) and primary cutaneous marginal zone lymphoma (PCMZL), which are typically indolent, and primary cutaneous diffuse large B-cell lymphoma, leg type (PCDLBCL-LT), which behaves aggressively [1,2]. PCFCL and PCMZL infrequently disseminate beyond the skin and are associated with excellent outcomes, with five-year disease-specific survival rates ≥95%, whereas PCDLBCL-LT carries a substantially worse prognosis with five-year survival below 60% [1-3]. Because systemic B-cell lymphomas can also secondarily involve the skin, current PCBCL guidelines emphasize careful clinicopathologic correlation and appropriate staging to distinguish primary cutaneous disease from cutaneous manifestations of systemic lymphoma [2-4].

Chronic lymphocytic leukemia/small lymphocytic lymphoma (CLL/SLL) is a distinct mature B-cell neoplasm that primarily involves blood, bone marrow, and lymph nodes but can, in some patients, infiltrate extranodal sites, including the skin. CLL is the most common adult leukemia in Western countries and typically affects older individuals; it is characterized by the clonal expansion of mature CD5-positive B cells with a highly variable clinical course [5-7]. Over the past decade, major advances in understanding CLL pathogenesis, including the identification of recurrent genetic alterations, clarification of clonal architectures, and insights into microenvironmental signaling, have led to refined risk stratification and the development of targeted therapies that have transformed management [5-7]. In parallel, genome-wide association studies have demonstrated a strong inherited component to CLL risk and have implicated dysregulation of multiple immunity-related genes, underscoring the central role of immune pathways in disease susceptibility [8].

Cutaneous involvement by CLL/SLL is uncommon but well-described and may occur at diagnosis or during the evolution of systemic disease. When CLL/SLL involves the skin, the resulting erythematous or violaceous papules, plaques, or nodules, often on the head, neck, or trunk, can be clinically indistinguishable from PCBCL [2-4]. Accurate classification, therefore, depends on integrating clinical findings with histopathology, immunophenotype, and staging studies to determine whether a cutaneous B-cell infiltrate represents a primary cutaneous lymphoma or secondary involvement by systemic CLL/SLL.

This report describes an elderly male with a solitary back nodule histologically and immunophenotypically consistent with CLL/SLL, highlighting the diagnostic challenges and management of cutaneous involvement by systemic B-cell lymphoma.

## Case presentation

**Figure 1:**
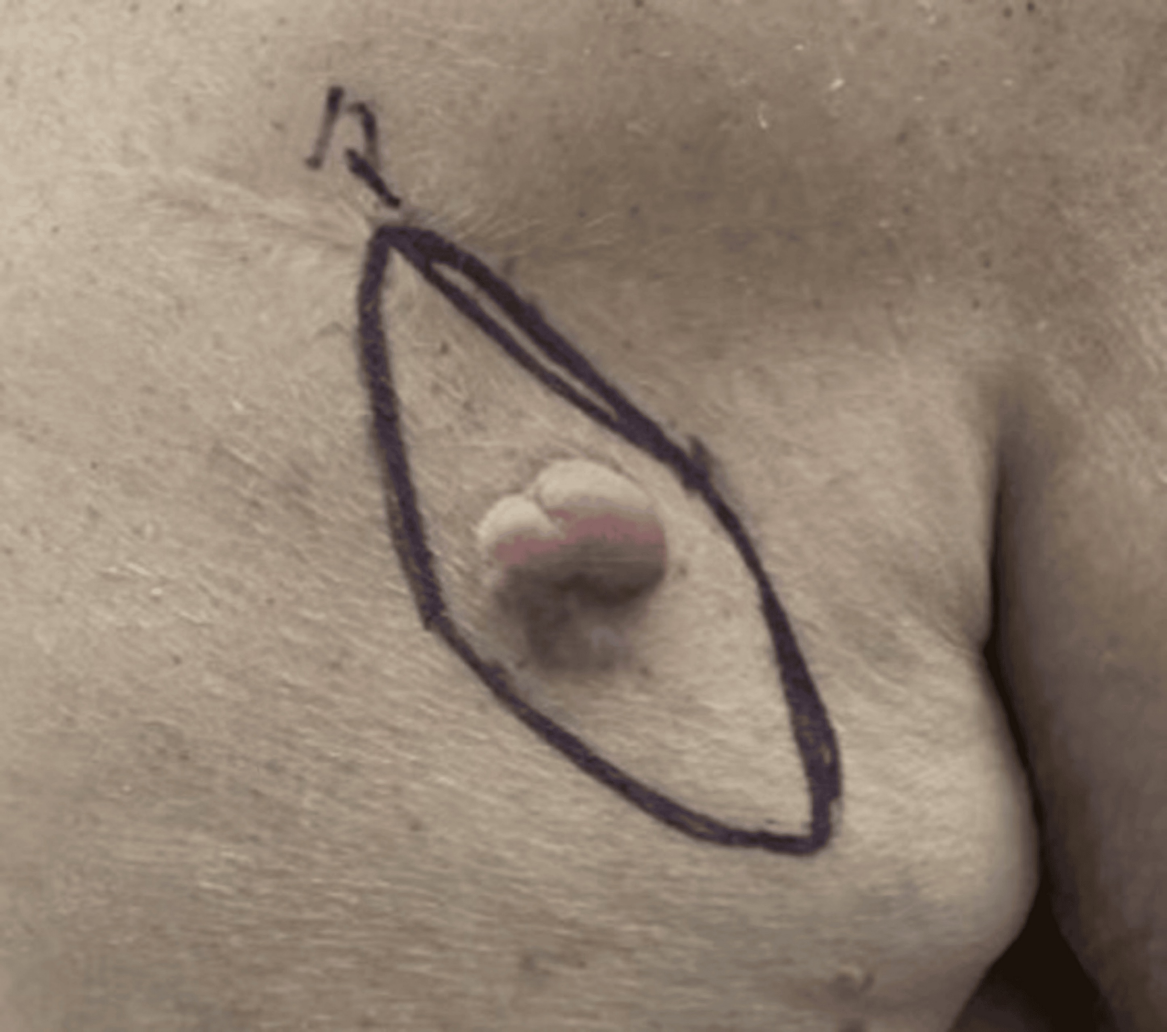
Pink, pedunculated papule/nodule on the right upper back representing cutaneous involvement by CLL/SLL. CLL/SLL: chronic lymphocytic leukemia/small lymphocytic lymphoma

**Figure 2:**
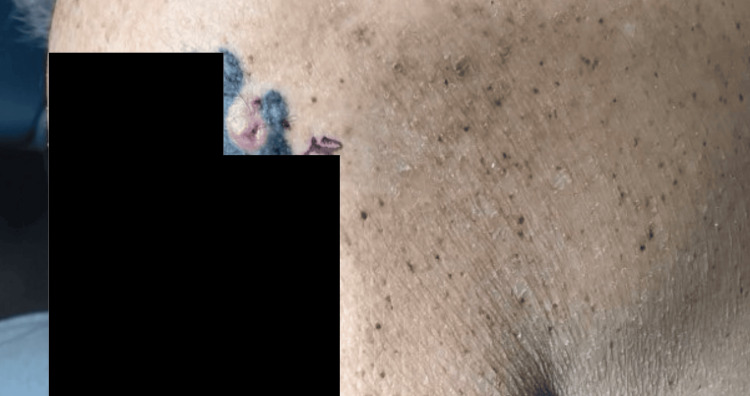
Papule on the left posterior shoulder characterized by a firm, pink, dome-shaped lesion; biopsy revealed a benign intradermal melanocytic (dermal) nevus without evidence of lymphoma.

An 85-year-old male presented in March of 2025 for evaluation of asymptomatic, enlarging skin lesions. The patient reported a slowly growing pink, pedunculated papule/nodule on the right upper back that had been present for many years and a papule on the left posterior shoulder. He denied prior treatment, pain, or bleeding. There was no personal or family history of melanoma or non-melanoma skin cancer. A focused examination of the back revealed a lesion on the right inferior lateral upper back and another on the left posterior shoulder. Shave biopsies were performed from both sites for histopathologic evaluation (Figures 1, 2).

At follow-up later that month, initial dermatopathology of the right upper-back shave biopsy demonstrated a dense diffuse infiltrate of relatively monomorphous lymphocytes. CD3 highlighted reactive T cells with a normal CD4:CD8 ratio, whereas CD20 highlighted the B-cell population. BCL2 was positive in most B cells, BCL6 highlighted focal pseudofollicles, and CD30 was negative. In situ hybridization for kappa and lambda light chains showed kappa predominance, and B-cell gene rearrangement studies (IgH and kappa) were positive, supporting a clonal B-cell proliferation interpreted as an atypical lymphocytic proliferation concerning low-grade B-cell lymphoma of marginal zone type. In contrast, the left posterior shoulder shave biopsy showed a benign intradermal melanocytic (dermal) nevus without evidence of lymphoma. Given the benign diagnosis, the left posterior shoulder nevus required no further treatment.

Because of the atypical infiltrate, the case was submitted for additional consultation. Subsequent review of the excisional specimen at a reference dermatopathology laboratory and at the National Institutes of Health demonstrated a nodular dermal infiltrate of small lymphocytes with a CLL/SLL immunophenotype (CD5+, CD23+, CD20+, BCL2+) and low Ki-67, leading to reclassification as cutaneous involvement by CLL/SLL. The patient had no palpable lymphadenopathy or evidence of systemic involvement on examination. He was referred to oncology for staging and treatment recommendations. A subsequent shave biopsy from the right inferior medial forehead performed for an additional lesion revealed sebaceous hyperplasia.

Oncology evaluation included a whole-body positron emission tomography (PET) scan and bone marrow biopsy. PET imaging showed no hypermetabolic lymphadenopathy or visceral disease. Bone marrow evaluation demonstrated a small population (3.5%) of monoclonal CD5- and CD23-positive B cells consistent with low-burden CLL/SLL involvement. Taken together, these findings indicated low-volume systemic disease with an isolated cutaneous manifestation. The patient remained asymptomatic without constitutional complaints or weight loss.

In June 2025, the patient returned for management of the biopsy-proven cutaneous B-cell lymphoma of the right upper back. After counseling regarding treatment options, surgical excision with primary closure was selected. Under local anesthesia with 1% lidocaine and epinephrine, a fusiform excision measuring 3.3 cm in total diameter was performed with 0.4 cm margins. The specimen was excised to the level of the adipose tissue and submitted for histopathologic analysis. Complex layered closure was completed with 3-0 Vicryl for deep subcutaneous layers and 3-0 Prolene for the epidermis. The final wound length measured 15 cm. Hemostasis was achieved without complication, and the site was dressed with petrolatum and a pressure bandage.

**Figure 3:**
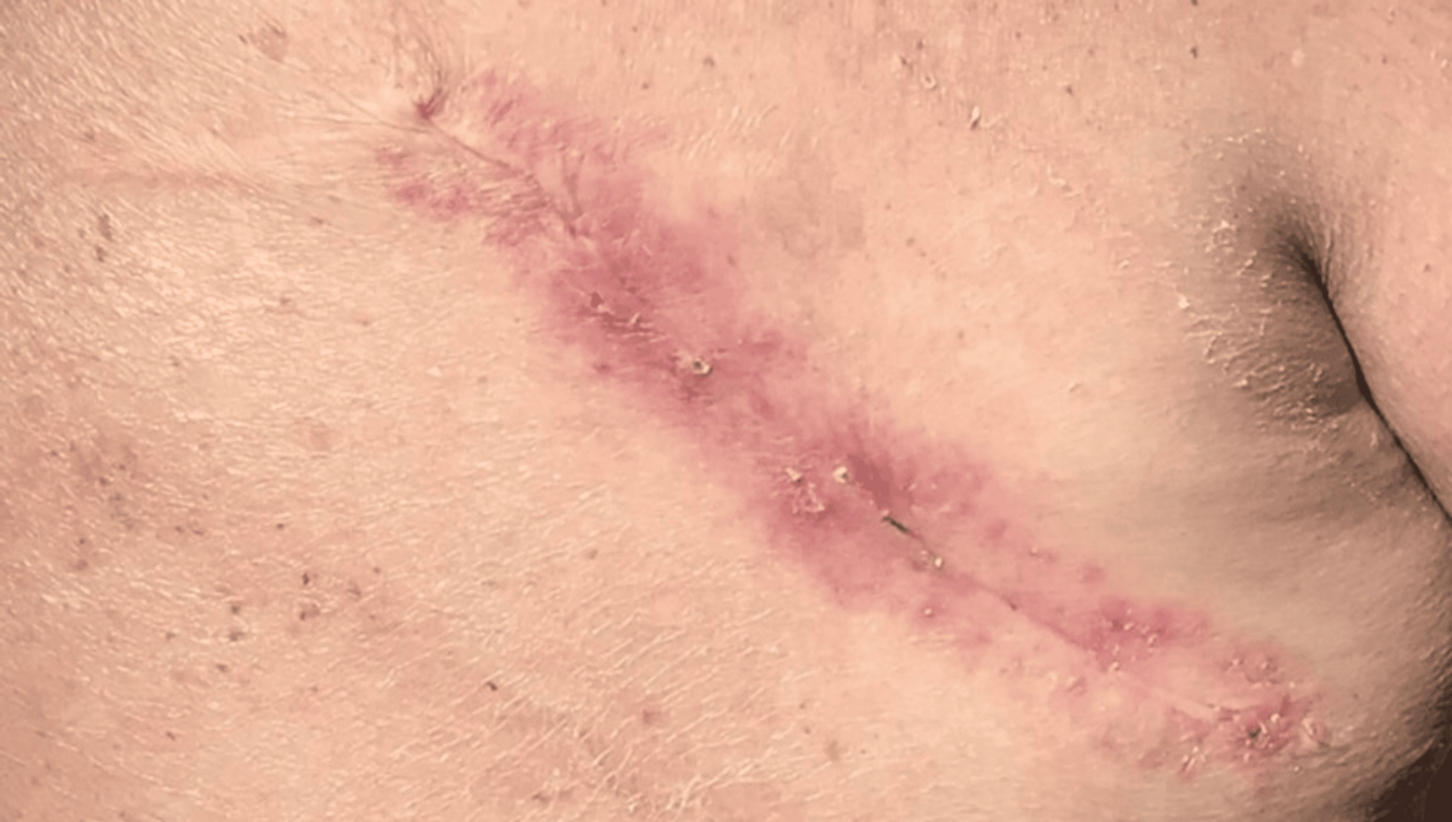
Well-healed linear scar on the right upper back two weeks after complex layered closure of the excised CLL/SLL nodule. CLL/SLL: chronic lymphocytic leukemia/small lymphocytic lymphoma

Two weeks later, sutures were removed from a clean, well-healed incision. No signs of infection, dehiscence, or recurrence were noted. At a full-body skin examination in July 2025, the surgical site remained stable, and there was no evidence of new or recurrent lesions (Figure 3). The patient continued follow-up with his oncologist every four months, with the most recent PET scan showing no disease progression.

## Discussion

CLL/SLL is a mature B-cell neoplasm characterized by small, monomorphic lymphocytes involving blood, bone marrow, and lymphoid tissues. Secondary cutaneous involvement by CLL/SLL is an uncommon but recognized manifestation and may present as papules, nodules, or plaques that closely resemble PCBCLs. Distinguishing these entities is important because staging, prognosis, and treatment strategies differ [1-5].

Current classification schemes emphasize a structured diagnostic approach that integrates clinical findings with histopathology, immunophenotyping, and staging studies such as PET/CT and bone marrow biopsy [6-8]. Ancillary molecular studies can further support the diagnosis; in this case, B-cell gene rearrangement analysis showed a clonal immunoglobulin heavy- and kappa light-chain gene rearrangement, whereas T-cell receptor gamma and beta rearrangements were negative, confirming a clonal B-cell process. In cutaneous lymphoid infiltrates, the presence of a CD5- and CD23-positive B-cell population with a CLL/SLL phenotype supports secondary involvement by systemic leukemia/lymphoma rather than a PCMZL, which more typically shows a plasma cell-rich infiltrate with light-chain restriction.

In our patient, the solitary reddish nodule on the upper back showed a dermal infiltrate of small lymphocytes with a CLL/SLL immunophenotype (CD5+, CD23+, CD20+, BCL2+) and low proliferative index. PET imaging did not reveal bulky nodal or visceral disease, and bone marrow demonstrated only low-level involvement, consistent with indolent CLL/SLL with cutaneous manifestation. Local excision of the cutaneous nodule resulted in a well-healed scar without clinical recurrence during follow-up, while the patient continued systemic surveillance with oncology.

This case highlights several teaching points. First, long-standing, slowly enlarging nodules in elderly patients warrant biopsy, even when asymptomatic or clinically suggestive of benign neoplasms. Second, cutaneous B-cell infiltrates with a CD5- and CD23-positive phenotype should prompt evaluation for CLL/SLL with appropriate staging, as these findings favor secondary involvement rather than primary cutaneous lymphomas. Finally, in patients with low-burden systemic disease and an isolated skin lesion, local excision may provide durable local control while systemic CLL/SLL is managed expectantly by hematology.

## Conclusions

This case illustrates cutaneous involvement by CLL/SLL presenting as a solitary, slowly enlarging back nodule in an elderly patient. Biopsy and detailed immunophenotyping were essential to distinguish this lesion from PCBCLs and to correctly classify it as CLL/SLL. Staging studies demonstrated low-burden systemic disease without bulky nodal or visceral involvement, and local excision of the cutaneous nodule led to a well-healed scar without local recurrence during dermatologic follow-up. Clinicians should maintain a high index of suspicion for secondary cutaneous involvement by systemic hematologic malignancies when evaluating chronic nodules in older adults, as accurate classification has important implications for staging, prognosis, and coordination of care with oncology.
